# Larger Physique as a Risk Factor for Infantile Appendicitis: A Retrospective Study

**DOI:** 10.3390/pediatric14010004

**Published:** 2022-01-04

**Authors:** Katsuhiro Nishimura, Keita Terui, Naoko Mise, Gen Matsuura, Mitsuyuki Nakata, Shugo Komatsu, Takeshi Saito, Tomoro Hishiki

**Affiliations:** 1 Department of Pediatric Surgery, Graduate School of Medicine, Chiba University, Chiba 260-8677, Japan; westwin0209@gmail.com (K.N.); mitchinakachi@gmail.com (M.N.); cbr@js5.so-net.ne.jp (S.K.); tomoro.hishiki@gmail.com (T.H.); 2 Department of Pediatric Surgery, Matsudo City General Hospital, Chiba 270-2296, Japan; rsc70508@nifty.com (N.M.); bzg13331@nifty.com (G.M.); 3 Department of Pediatric Surgery, Chiba Children’s Hospital, Chiba 266-0007, Japan; takksa5@gmail.com

**Keywords:** appendicitis, body weight, diagnosis, abdominal pain, pediatric emergency medicine

## Abstract

The clinical features and risk factors of acute appendicitis in infants are unclear. Our aim was to evaluate the association between anthropometrics and the occurrence of infantile appendicitis. This was a retrospective study of infants (<6 years of age) and school-age children (6–10 years of age) of Asian ethnicity who required hospitalization for appendicitis at our two participating institutions between 2004 and 2018. The Z-score for height, body weight, and body mass index (BMI) was compared between the two groups, as well as between patients presenting with perforated and non-perforated appendicitis. The analysis included data from 73 infants and 362 school-age children. Z-scores were greater in infants than in school-age children for height (0.37 versus −0.03, *p* = 0.003) and body weight (0.12 versus −0.36, *p* = 0.023), with no between-group difference for the Z-score of BMI. There was no difference in Z-scores for height, weight, and BMI between the perforated and non-perforated appendicitis infant groups. Infants presenting with acute appendicitis were characterized by a larger physique but with normal proportion. This trend was not observed in school-age children. Therefore, larger infants presenting with abdominal pain should be screened for appendicitis.

## 1. Introduction

Acute appendicitis is a common pediatric emergency, with a prevalence rate of 1–8% among children presenting with acute abdominal pain [1] and a prevalence rate of 2–9% among children <6 years of age [1,2,3]. The high incidence of perforating appendicitis among pre-school children is concerning [4], with an incidence rate of 43–74% among children <5 years of age [2,4,5,6]. Lin and Lee [7] reported on a case of appendicitis-related mortality in a 5-month-old child, which underlines the importance for pediatric primary-care physicians to screen for appendicitis among a large number of infants and young children who present with acute abdominal pain. However, the diagnosis of infantile appendicitis is not easy due to difficulties in confirming presenting symptoms. Moreover, the symptoms of infantile appendicitis, which include abdominal pain, fever, poor appetite, and diarrhea [6], are not specific, as they overlap with symptoms of enteritis and constipation, which are the most common causes of abdominal pain presenting in primary-care pediatric settings. Thus, specific risk factors for infantile appendicitis are needed, particularly as we consider the high misdiagnosis rate of infantile appendicitis, which ranges between 44 and 57% among pre-school children [5,8]. Based on our clinical experiences, we have more often observed that appendicitis occurs more frequently among children with a large body size. Accordingly, the purpose of our study was to evaluate the possible association between anthropometrics and appendicitis among infants and school-age children.

## 2. Materials and Methods

### 2.1. Study Population

Eligible patients were those of Asian ethnicity and <11 years of age with a diagnosis of appendicitis who required hospitalization at either of our two participating institutions between January 2004 and December 2018. Patients with carcinoid-induced appendicitis were excluded, as well as those for whom anthropometric measurements were not obtained on admission. For patients with repeated admissions for appendicitis, only the data of the first admission were used. Patients managed with either surgical or non-surgical treatment were included.

### 2.2. Diagnostic Criteria

For patients who underwent surgical treatment, the diagnosis of appendicitis was confirmed by macroscopic assessment and histological findings. For those treated conservatively, the diagnosis was based on imaging assessment using ultrasound (US) or computed tomography (CT), with a swollen appendix of ≥6 mm considered as a positive finding.

### 2.3. Variables

Medical records were retrospectively reviewed, with the following data extracted for analysis: age, sex, height and body weight on admission, and severity of appendicitis (namely perforated or non-perforated). Perforation was diagnosed by direct observation in surgical patients and by findings of appendiceal abscess or massive ascites on US or CT images in non-operative patients. The body mass index (BMI) was calculated as the weight/height^2^ (kg/m^2^). The Z-score of the height and body weight was calculated relative to population norm references by age and sex obtained from the national databases for 12,426 infants and 695,600 school-age children [9,10].

### 2.4. Statistical Analysis

Continuous variables are expressed as the median and interquartile range (IQR), and categorical variables as the count and percentage. For analysis, patients were divided into two groups according to their age at the time of admission, namely the infants group (<6 years of age) and the school-age children (≥6 years of age). Between-group differences were evaluated using Fisher’s exact probability test or the chi-squared test, as appropriate, for categorical variables and the Mann–Whitney U test for continuous variables. The association between the age and Z-score of the height, body weight, and BMI, as well as between the Z-scores and the severity of appendicitis was evaluated using Pearson’s correlation coefficient or Spearman’s rank correlation, as appropriate for the data type, and expressed using the corresponding correlation coefficient (r). *p*-values of <0.05 were considered significant.

All statistical analyses were performed using EZR (Saitama Medical Center, Jichi Medical University, Saitama, Japan), which is a graphical user interface for R (The R Foundation for Statistical Computing, Vienna, Austria).

## 3. Results

### 3.1. Patients

A total of 580 patients were eligible for our study; of these, 145 were excluded due to a lack of anthropometric data on admission, wherein four were of non-Asian ethnicity and one patient had a diagnosis of carcinoid-induced appendicitis. Following this exclusion of 25% of the prospective cohort, 435 patients met our inclusion criteria and formed our study group, including 73 infants and 362 school-age children. Demographic data for our study group are reported in Table 1, with anthropometric data at the time of admission presented in Table 2.

### 3.2. Comparison of Z-Scores between Infants and School-Age Children

**Figure 1:**
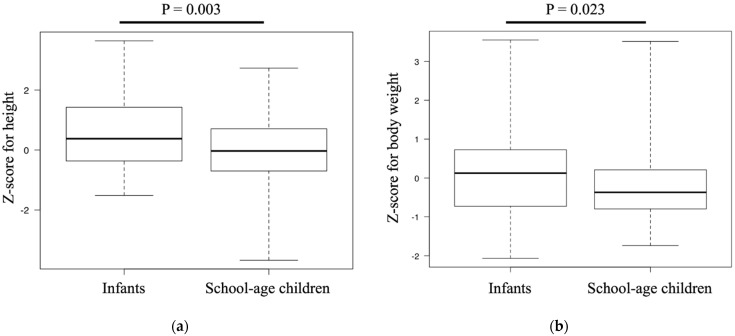
Comparison of Z-scores between the infant and school-age groups: (**a**) Z-score for height and (**b**) Z-score for body weight. The box plots show the interquartile range as well as the maximum and minimum values.

The Z-score for height was significantly greater among infants than among school-age children (0.37 (−0.36–1.42) versus −0.03 (−0.69–0.70), *p* = 0.003; Figure 1a). Similarly, the Z-score for body weight was significantly greater in infants than in school-age children (0.12 (−0.72–0.72) versus −0.36 (−0.79–0.20), *p* = 0.023; Figure 1b). There was no difference in the Z-score for the BMI between the two groups (−0.21 (−0.81–0.33) versus −0.29 (−0.97–0.42), *p* = 0.861).

### 3.3. Correlation between Age and Z-Score in Infants

**Figure 2:**
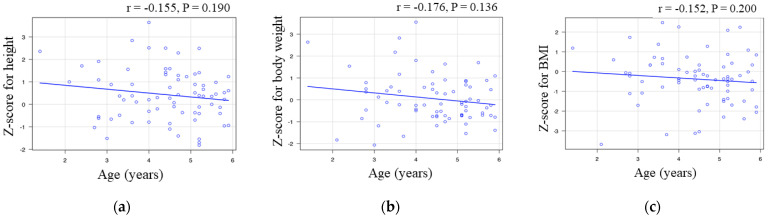
Correlation between age and the Z-scores in the infant group: (**a**) age versus Z-score for height; (**b**) age versus Z-score for body weight; and (**c**) age versus Z-score for body mass index. The correlation coefficient “r” is included.

Among infants, there was no correlation between age and the Z-score for height (r = −0.155, *p* = 0.19; Figure 2a), body weight (r = −0.176, *p* = 0.136; Figure 2b), and BMI (r = −0.152, *p* = 0.2; Figure 2c).

### 3.4. Comparison of Z-Scores between Patients Perforated and Non-Perforated Appendicitis

The rate of perforated appendicitis was higher among infants (n = 54, 74%) than among school-age children (n = 120, 33%; *p* < 0.001). There was no difference in Z-scores between infants with and without perforated appendicitis: height, 0.42 (−0.18–1.10) versus 0.36 (−0.46–1.26), *p* = 0.860; body weight, −0.12 (−0.62–0.52) versus −0.19 (−0.72–0.65), *p* = 0.797; and BMI, −0.36 (−0.86–0.33) versus −0.36 (−1.07–0.33), *p* = 0.787.

## 4. Discussion

This study investigated the possible association between anthropometrics and appendicitis among infants and school-age children. The principal findings of our study were as follows. First, infants with acute appendicitis were larger in height and weight than the norm-reference for the general Asian population by age and sex; this trend was not observed in the school-age group. Second, age was not correlated to the Z-score for height, weight, or BMI in infants. Third, the severity of appendicitis was not related to the Z-score for height, weight, or BMI.

There are several clinical scores available to predict the risk of appendicitis in children with acute abdominal pain, with the Pediatric Appendicitis Score (PAS) being commonly used. The PAS predicts the risk of appendicitis based on an assessment of the following eight parameters [11]: (1) cough/percussion/hopping tenderness of the right lower quadrant of the abdomen; (2) anorexia; (3) pyrexia; (4) nausea/emesis; (5) tenderness over the right iliac fossa; (6) leukocytosis; (7) polymorphonuclear neutrophilia; and (8) pain migration. However, the PAS may underestimate the severity of appendicitis in children younger than 4 years of age [12]. This is clinically significant considering the high risk for perforated appendicitis among young patients as a function of increasing symptom duration [13]. Additionally, clinical symptoms of infantile appendicitis are difficult to differentiate from those of enteritis or constipation, which are conditions that are more frequently encountered in pediatric practice [6]. Regarding the association between anthropometrics and the risk of appendicitis, Ramos and Nieves-Plaza [14] reported that a perforated appendix is likely to occur in obese children. However, they did not find a significant association between BMI and the incidence of perforation. The findings of our study indicate that, although anthropometrics influence the risk for acute appendicitis, they are not associated with the severity of appendicitis.

The association between anthropometrics and the risk of appendicitis is unclear; however, genetic factors may play a role. In their recent genomic study of appendicitis in adults, Kristjansson et al. [15] identified an association between a sequence variant at 4q25 near *PITX2* and the risk of appendicitis. *PITX2* is also known as *Pituitary homeobox 2*, which may play a role in the proper localization of asymmetric organs [16]. However, an association between *PITX2* and growth has not previously been reported. Based on current evidence, it is unclear whether there is an association between a genetic factor, anthropometrics, and the risk of appendicitis in children. Diet may also be an influencing factor. It has been suggested that a decrease in dietary fiber and ingestion of refined carbohydrates are significant risk factors for appendicitis [17]. Dietary fiber deficiency causes the gut microbiota to use glycoproteins in the host’s intestinal mucus as a source of nutrients, leading to a disruption of the colonic mucosal barrier and enteritis [18]. Diet may also affect anthropometric characteristics, with a larger physique being influenced by the amount of food consumption and dietary content. The link between anthropometrics and appendicitis in children could be mediated by the effects of diet on the intestinal flora, which would contribute to the pathogenesis of appendicitis. Further evidence is needed to test this hypothesis. A plausible association between intestinal flora has recently been reported [19]; specifically, this was an association between the oral microbiome, fusobacteria, and appendicitis. Fusobacteria are oral pathogens of periodontic diseases associated with extra-oral diseases, such as inflammatory bowel disease and colorectal cancer [20]. Swidsinski et al. [19] identified fusobacteria as a specific component of appendiceal epithelial and submucosal invasion in patients with appendicitis, with a positive correlation between the presence of fusobacteria in mucosal lesions and the severity of appendicitis. Zhong et al. [21] reported an increase in fusobacteria and a decrease in bacteroides among patients with appendicitis compared to those in a control group without appendicitis. In their study, Zhong et al. further reported that appendiceal flora in patients with appendicitis contained variable amounts of other oral taxa not found among individuals without appendicitis. Current evidence therefore indicates that appendicitis may be a polymicrobial infection associated with resident bacteria in the oral cavity, such as fusobacteria. The pathway by which oral flora reach the appendix remains to be defined. A hematogenous pathway has been proposed, with resident bacteria in the oral cavity entering the bloodstream during teething in infants. This pathway describes the second increase in the risk of appendicitis around the age of 7 years, when permanent teeth erupt, and in the early 20s when full dentition is completed. This hypothesis would also explain the lower incidence of infantile appendicitis.

The limitations of our study need to be acknowledged. First is the retrospective design of our study, with a relatively small study sample, which limits the inferencing of any causality. Second, variables relevant to nutrition, social status, and parents’ anthropometric measurements were not considered in the analysis. Third, only infants and children of Asian ethnicity were included, requiring validation for other ethnicities. Finally, there is a need for a larger validation study in a greater number of pediatric emergency departments.

## 5. Conclusions

Infants with acute appendicitis tend to be larger than the norm-reference population. Therefore, it would be warranted to screen larger infants and school-age children presenting to the emergency department for acute abdominal pain for appendicitis.

## Figures and Tables

**Table 1:** Demographic data of infants and school-age children.

	Infants Group (n = 73)	School-Age Group (n = 362)	*p*-Value
Median age, years (range)	4.6 (1.4–5.9)	9.1 (6.0–10.9)	
Male, n (%)	42 (57)	241 (66)	0.179
Number of patients with a perforated appendix, n (%)	54 (74)	120 (33)	<0.001 *
Surgery, n (%)	53 (72)	299 (82)	0.051

* *p* < 0.05.

**Table 2:** Anthropometric data of patients on admission by age.

Age (years)	Patients, n (%)	Height (cm)	Body Weight (kg)	BMI (kg/m^2^)
1	1 (0.2)	86.2	13.2	17.7
2	7 (1.6)	91.8 ± 3.6	12.8 ± 1.6	15.1 ± 1.7
3	12 (2.8)	96.8 ± 5.1	14.6 ± 2.5	15.4 ± 1.7
4	25 (5.8)	105.7 ± 5.1	16.6 ± 2.2	14.8 ± 1.3
5	28 (6.4)	109.8 ± 4.8	18.0 ± 2.3	14.9 ± 1.8
6	45 (10.3)	116.8 ± 4.7	20.8 ± 3.8	15.1 ± 1.9
7	58 (13.3)	122.0 ± 6.5	23.4 ± 4.4	15.7 ± 2.5
8	69 (15.9)	128.0 ± 6.0	26.0 ± 5.5	15.7 ± 2.5
9	93 (21.4)	133.0 ± 6.1	29.3 ± 5.5	16.5 ± 2.3
10	97 (22.3)	139.1 ± 5.0	33.7 ± 5.9	17.3 ± 2.3

Anthropometric data are shown as the mean ± SD. BMI, body mass index.

## Data Availability

The datasets of the current study are available from the corresponding author upon reasonable request.
